# Supplementary material for: Multiparametric quantitative phase imaging for real-time, single cell, drug screening in breast cancer
Source: Commun Biol. 2022 Aug 8;5:794. doi: 10.1038/s42003-022-03759-1 (PMC9360018; doi:10.1038/s42003-022-03759-1)
Supplement: Supplementary file 2 — Supplementary Information [file 42003_2022_3759_MOESM2_ESM.pdf]

Supplemental Material for

Multiparametric quantitative phase imaging for real-time, single cell, drug screening in breast cancer

Edward R. Polanco[1], Tarek E. Moustafa[1], Andrew Butterfield[2,3], Sandra D. Scherer[2,3], Emilio Cortes-Sanchez[2,3], Tyler Bodily[1], Benjamin T. Spike[2,3], Bryan E. Welm[2,4], Philip S. Bernard[2,5,6], Thomas A. Zangle[1,2*]

[1]Department of Chemical Engineering, University of Utah, Salt Lake City, UT, USA
[2]Huntsman Cancer Institute, University of Utah, Salt Lake City, UT, USA
[3]Department of Oncological Sciences, University of Utah, Salt Lake City, UT, USA
[4]Department of Surgery, University of Utah, Salt Lake City, UT, USA
[5]Department of Pathology, University of Utah, Salt Lake City, UT, USA
[6]ARUP Institute for Clinical and Experimental Pathology, Salt Lake City, UT, USA

*tzangle@chemeng.utah.edu

The PDF file includes:

Supplementary Tables S1-S3
Supplementary Figures S1-S19

Table S1. Cell lines and receptor status	
**Cell line**	**Receptor status**
MCF-7	ER+/PR+/HER2-
MDA-MB-231	ER-/PR-/HER2-
BT-474	ER-/PR+/HER2+

Table S2. List of microscope components		
**Part**	**Supplier**	**Model/part number**
Arduino Metro M4	Adafruit	3382
0.8", 8x8 LED array	Adafruit	870
High speed *xy* stage	Thorlabs	MLS203
10x Olympus PLAN Objective	Thorlabs	RMS10X
z-translation stage	Thorlabs	SM1Z
Flexible drive shaft	McMaster-Carr	3135K15
Sparkfun 2-phase stepper motor	Mouser	474-ROB-10846
25 mm right angle prism	Thorlabs	PS911
30 mm cage cube	Thorlabs	CCM1-4ER
SM1 cage plate adaptor	Thorlabs	LCP6X
SM2 cage plate	Thorlabs	LCP01
Tube lens (f = 200 mm)	Thorlabs	ITL200
ITL200 adaptor	Thorlabs	SM2A20
∅2" lens tube	Thorlabs	SM2L2
∅1" lens tube	Thorlabs	SM1L2
Grasshopper3 camera	Teledyne-FLIR	GS3-U3-23S6M-C

Table S3. List of compounds		
**Drug name**	**Abbreviation**	**Target**
5-Fluorouracil	5FU	DNA-RNA synthesis
Carboplatin	carb	DNA synthesis
Docetaxel	doc	Microtubule
Doxorubicin	dox	DNA topoisomerase II
Fulvestrant	fulv	Estrogen receptor
Lapatinib	lap	EGRF/HER2
Palbociclib	palb	CDK4/6
4-hydroxy-Tamoxifen	4HT	Estrogen receptor
Vinblastine	vin	Microtubule

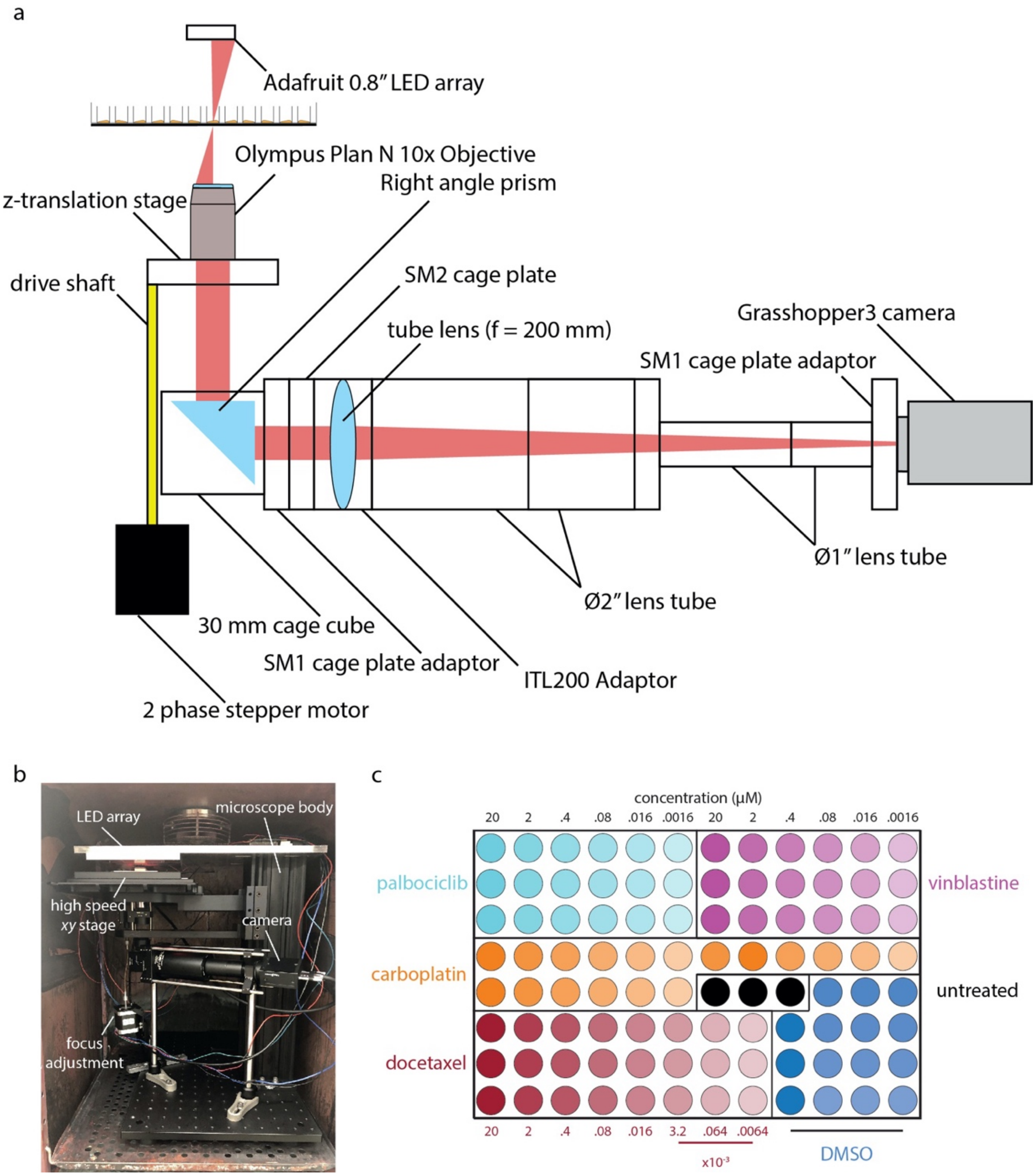

Supplementary Figure S1: (a) Diagram of the optical system showing the arrangement of elements found in Table S2. (b) Photograph shows implementation of DPC QPI microscope placed inside a cell culture incubator for temperature and CO_2 control throughout the duration of the experiment. (c) Plate map showing how therapies for drug panel B are organized. Docetaxel uses an expanded range of concentrations to capture the typically low EC_{50} for this drug, noted in maroon below the panel.

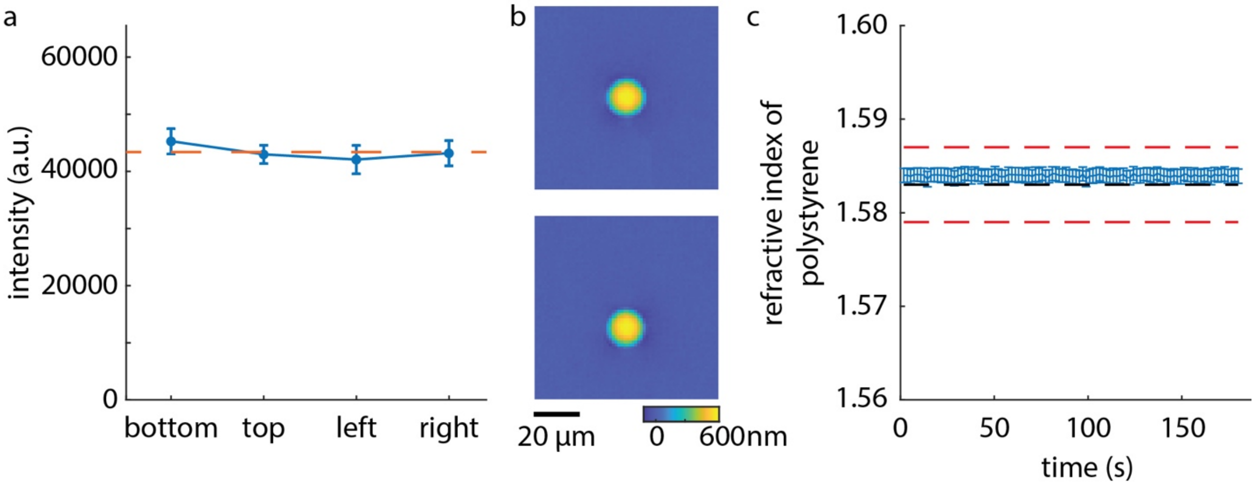

Supplementary Figure S2: Calibration data for QPI microscope. (a) Mean intensity data for the LED array with each half of the array illuminated as during cell growth experiments, error bars represent the standard deviation of intensity. Dashed green line is plotted at an intensity of 43,400 a.u., the mean intensity of all 4 halves of the LED array. (b) Example images of polystyrene beads embedded in NOA73 used to calibrate phase shift measurements. (c) Measured refractive index of polystyrene beads embedded in NOA73 during repeated QPI measurements over time. Blue line shows the mean refractive index measured across all the beads (n = 20), error bars show the standard error of the mean. Black line is at n = 1.583, the previously reported refractive index for polystyrene, and red lines show the uncertainty in the known refractive index of the NOA73 matrix.

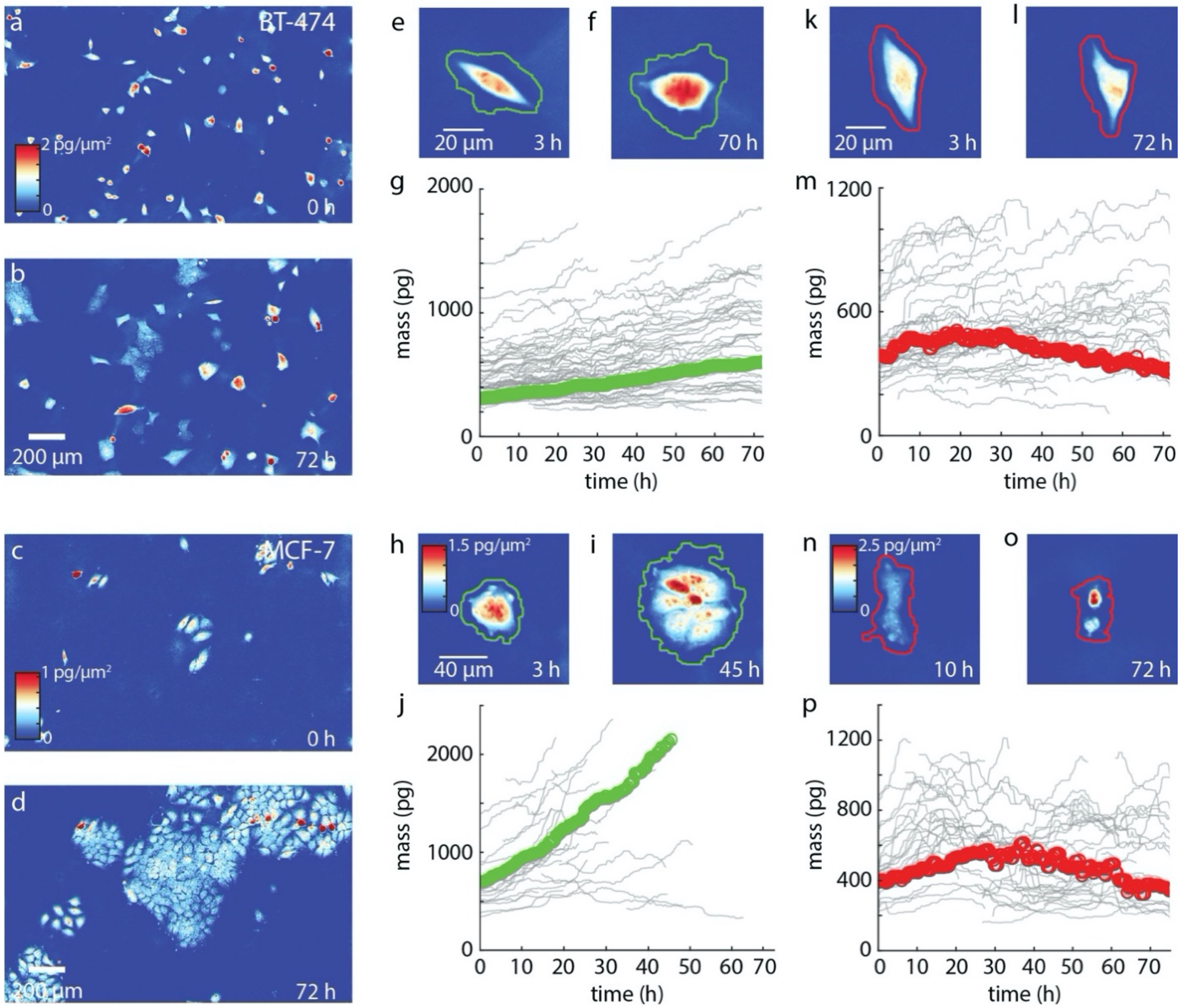

Supplementary Figure S3: QPI measures the drug sensitivity and temporal dynamics of cell clusters. (a) Field of view of BT-474 cells treated with DMSO at the beginning of a 72 h experiment (b) and at the end of the experiment where they have grown into clusters. (c) An example of a well containing MCF-7 cells at the beginning of the experiment from the DMSO control. (d) By the end of the experiment, the MCF-7 cells have grown to fill most of the field of view. (e) Cluster of 3 BT-474 cells from the DMSO solvent control that grow into (f) a larger cluster of 6 cells that are segmented together (green) to measure the growth of the cluster. (g) The individual mass measurements for this cluster are plotted showing robust growth. (h) A small cluster of MCF-7 cells treated with DMSO at the beginning of the experiment, (i) grows into a larger cluster by the 45 h timepoint. (j) Measuring the mass of this cluster demonstrates robust growth. (k) An example of a small cluster of BT-474 cells exposed to 2 μM of doxorubicin at the beginning of the experiment. (l) At the end of the experiment, the cluster has not grown noticeably in size. (m) The clusters mass is tracked over time shows the cluster is losing mass. (n) However, MCF-7 cells exposed to 20 μM fulvestrant at the 10 h (o) and 72 h timepoint appear to shrink in size over the course of the experiment. (p) Plotting the mass over time for this small cluster, shows that they grow slowly at the beginning and end the experiment with a lower mass than they started with.

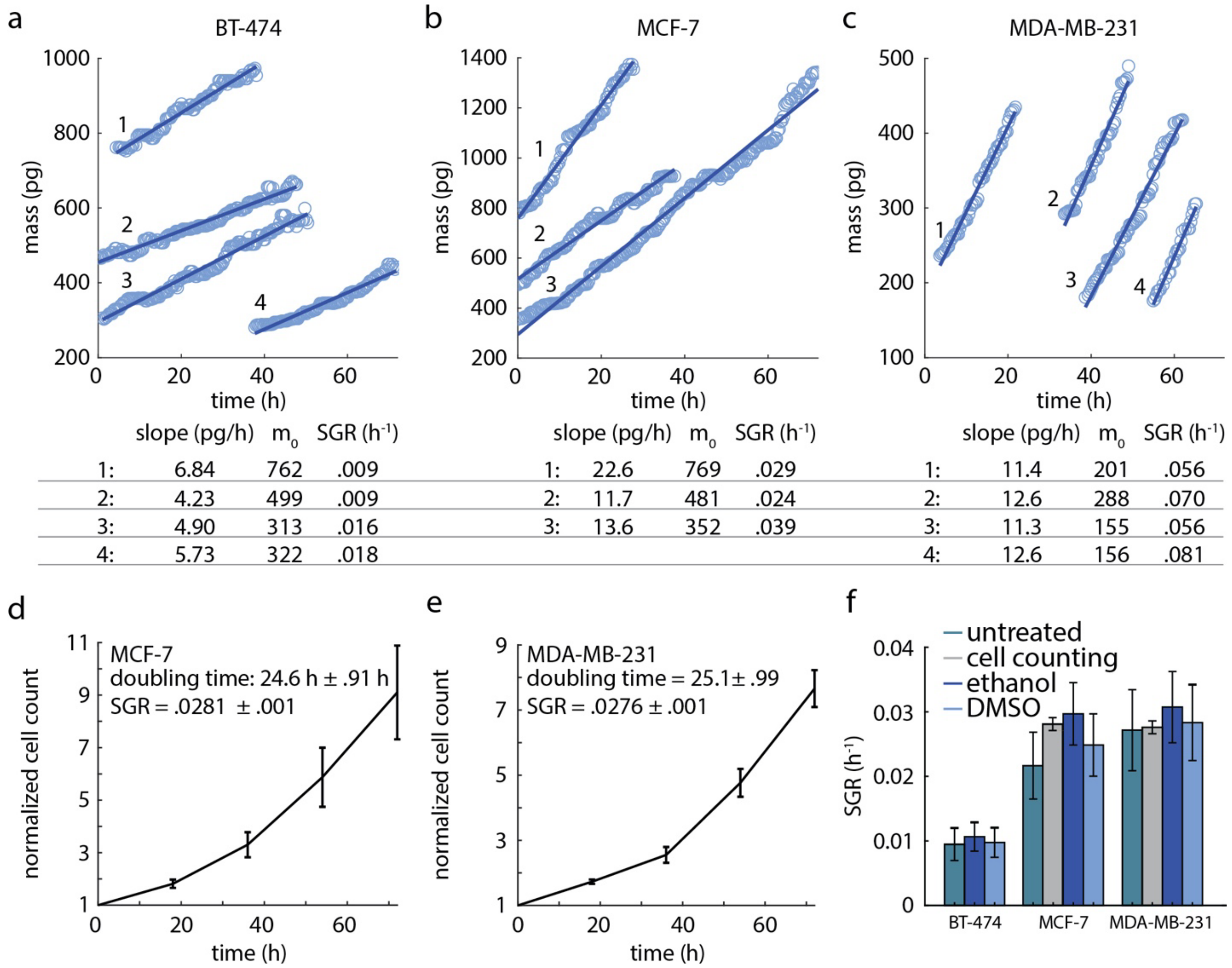

	BT-474 slope (pg/h)	BT-474 m_0	BT-474 SGR (h^{-1})	MCF-7 slope (pg/h)	MCF-7 m_0	MCF-7 SGR (h^{-1})	MDA-MB-231 slope (pg/h)	MDA-MB-231 m_0	MDA-MB-231 SGR (h^{-1})
1:	6.84	762	.009	22.6	769	.029	11.4	201	.056
2:	4.23	499	.009	11.7	481	.024	12.6	288	.070
3:	4.90	313	.016	13.6	352	.039	11.3	155	.056
4:	5.73	322	.018				12.6	156	.081

Supplementary Figures S4: Linear relationship between mass and growth. (a-c) Mass over time plots for BT-474, MCF-7, and MDA-MB-231 cells shows a linear fit to measure the growth rate of individual cells. (d) Proliferation measurements of MCF-7 cells treated with ethanol shows a doubling time of approximately 24.6 h, which corresponds to an exponential growth constant of 0.0281 h^{-1}. (e) Proliferation measurements of ethanol treated MDA-MB-231 cells shows a doubling time of 25.1 h, which corresponds to an exponential growth constant of 0.0276 h^{-1}. (f) Growth rate measured using cell counting shows similar results to growth rate measured using QPI. Error bars in (d), (e), (f) show the standard error of the mean.

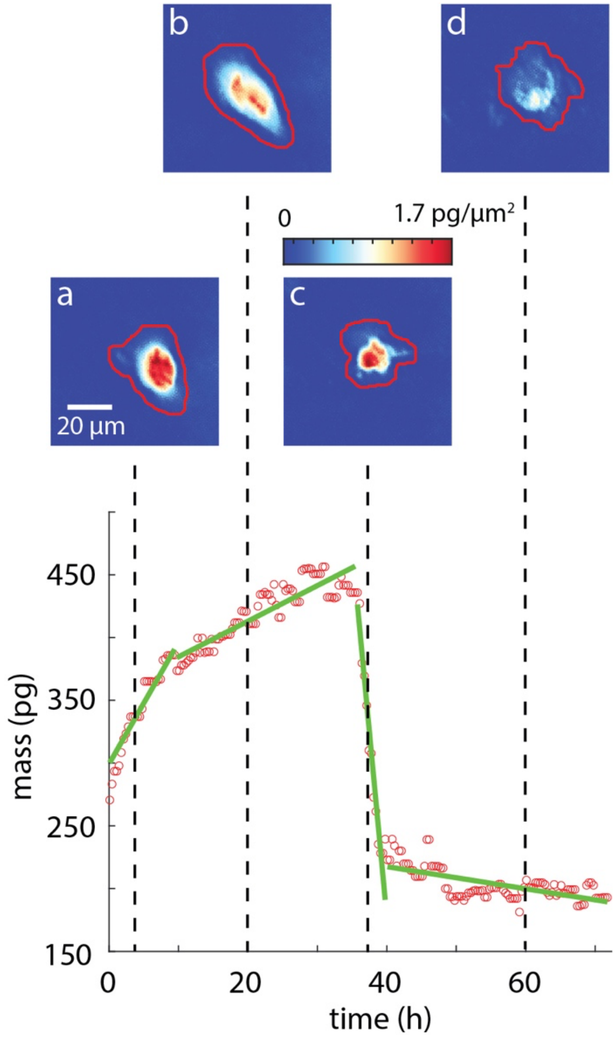

Supplementary Figure S5: Temporal dynamics of drug response for a single MDA-MB-231 cell during exposure to 2 µM doxorubicin. (a) Cell initially exhibits robust growth (SGR = 0.0358 h^{-1}). (b) After about 13 h of imaging the growth rate of the cell abruptly declines (SGR =0.0105 h^{-1}) for the following 19 h. (c) The cell then rapidly loses mass (SGR = -0.217 h^{-1}) at approximately 40 h post-exposure during the active stages of cell death. (d) The cell then continues losing mass (SGR = -0.0033 h^{-1}) throughout the rest of the experiment.

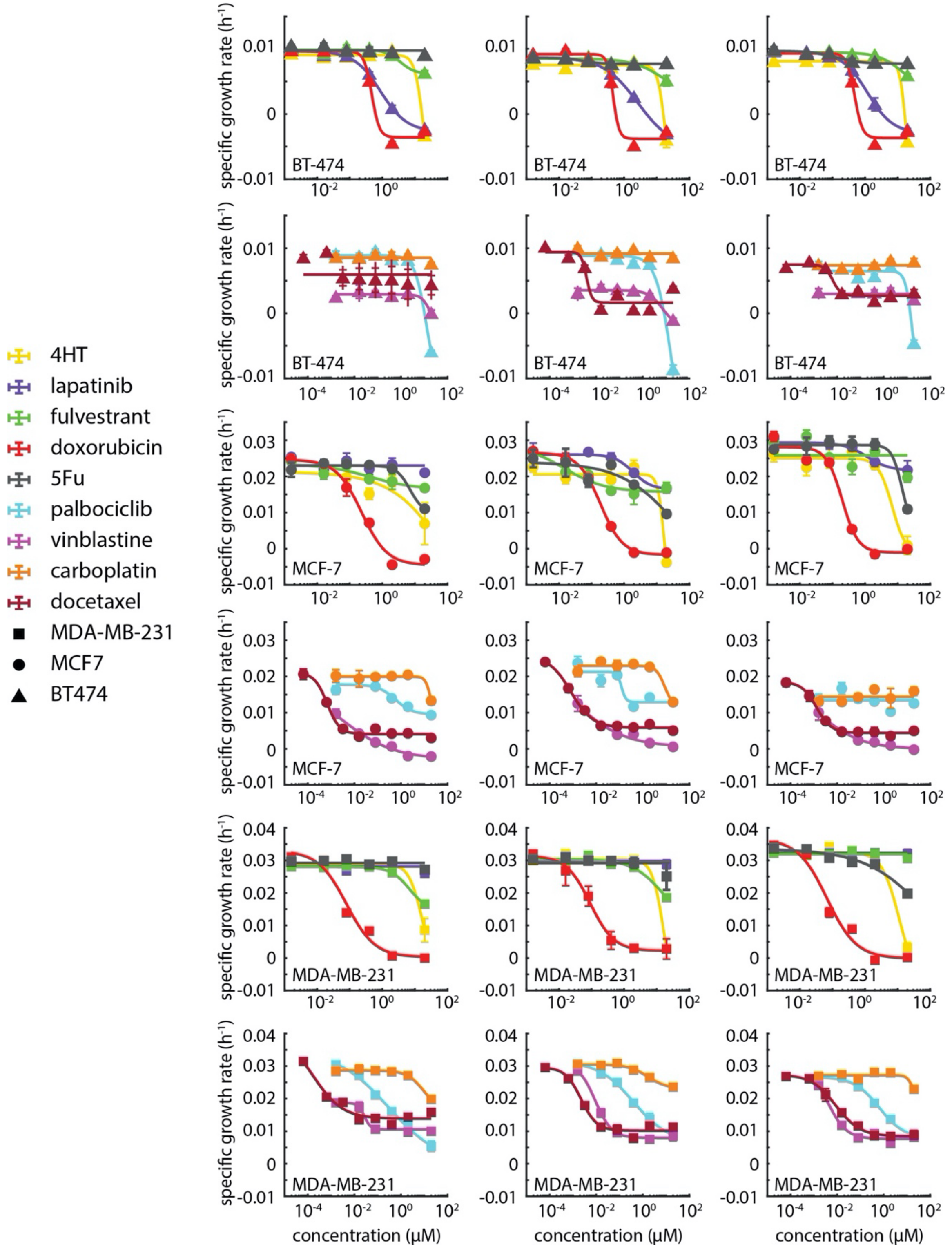

Supplementary Figure S6: Dose response measurements for all cell lines and drug combinations. Dose response plots show mean specific growth rate as a function of concentration. A 4-parameter Hill curve is fit to each dose response plot to extract the depth of response and EC_{50} for each cell/drug pair. A flat line represents no response due to failure of the F-test to reject the null hypothesis that the logistic fit is indistinguishable from a flat line.

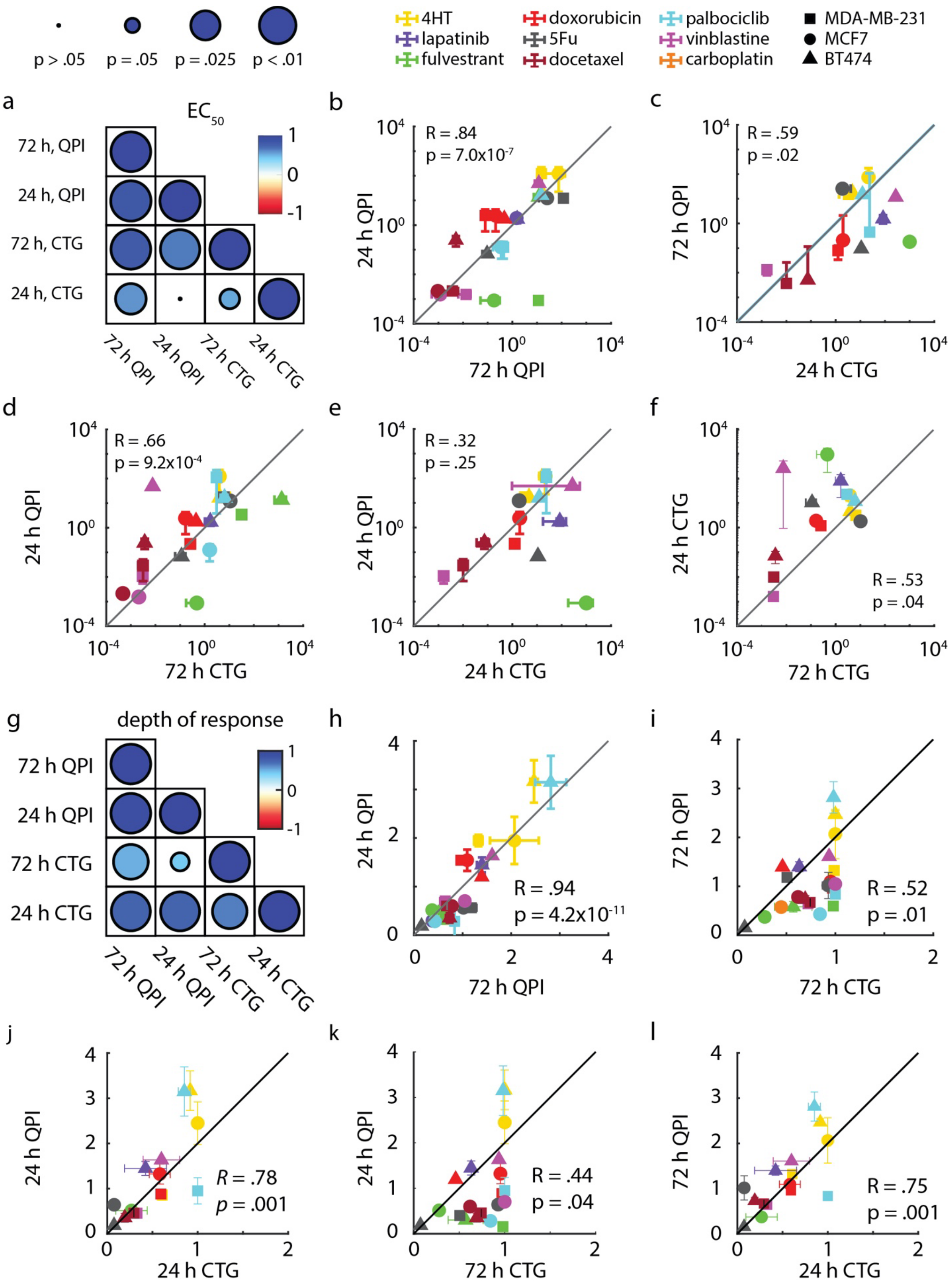

p > .05
p = .05
p = .025
p < .01
4HT
lapatinib
fulvestrant
doxorubicin
5Fu
docetaxel
palbociclib
vinblastine
carboplatin
MDA-MB-231
MCF7
BT474
a
EC50
72 h, QPI
24 h, QPI
72 h, CTG
24 h, CTG
72 h QPI
24 h QPI
72 h CTG
24 h CTG
b
R = .84
p = 7.0x10-7
c
R = .59
p = .02
d
R = .66
p = 9.2x10-4
e
R = .32
p = .25
f
R = .53
p = .04
g
depth of response
h
R = .94
p = 4.2x10-11
i
R = .52
p = .01
j
R = .78
p = .001
k
R = .44
p = .04
l
R = .75
p = .001

Supplementary Figure S7: 24 h QPI data predicts results in 72 h CTG and QPI experiments. (a) Correlation map of EC_{50} for both QPI and CTG experiments. Color indicates correlation (blue high, red low) and symbol size indicates *p*-value. (b) EC_{50} values determined from 72 h QPI and from the first 24 h of QPI data are strongly correlated ($R = .84$, $p = 7x10^{-7}$). (c) EC_{50} values from 24 h QPI and 72 h CTG are strongly correlated ($R = .66$, $p = 9.2x10^{-4}$). (d) EC_{50} values measured in 72 h using QPI and 24 h CTG are moderately correlated ($R = .59$, $p = .02$). (e) EC_{50} values obtained using 24 h of QPI data and 24 h CTG are not well correlated ($R = .32$, $p = .25$). (f) EC_{50} values measured using 24 h CTG and 72 h CTG are moderately correlated ($R = .53$, $p = .04$). (g) Correlation matrix of DoR measured using both QPI and CTG experiments. Color indicates correlation (blue high, red low) and symbol size indicates *p*-value. (h) DoR values measured in first 24 h of QPI and 72 h QPI are strongly correlated ($R = .71$, $p = 1.4x10^{-4}$). (i) DoR determined using 72 h QPI and 24 h CTG are not well correlated ($R = .48$, $p = .08$). (j) The DoR values determined in the first 24 h of QPI and 24 h CTG are strongly correlated ($R = .71$, $p = 3.0x10^{-3}$). (k) DoR measurements from the first 24 h of QPI and 72 h CTG are moderately correlated ($R = .5$, $p = .02$). (l) DoR measurements obtained using 72 h of QPI and 72 h CTG are moderately correlated ($R = .5$, $p = .01$). The gray line is y = x. The black line is y = 2x. Error bars show SEM.

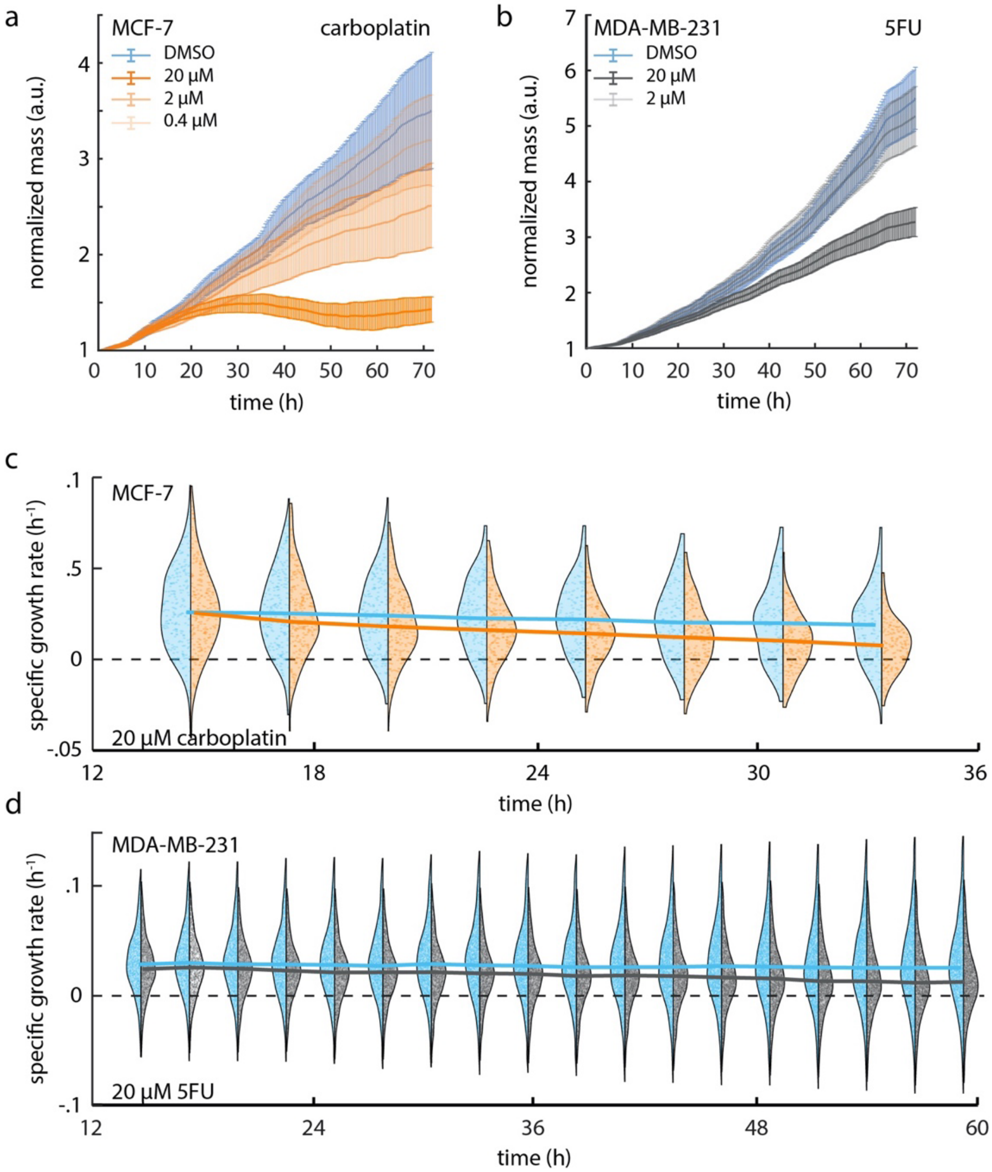

Supplementary Figure S8: Dynamic response plots for MCF7 and MDA-MB-231. (a) Average mass plotted against time, for carboplatin treated MCF-7 cells. Mass is normalized by the initial mass of each cell in the population. Dark orange represents 20 µM carboplatin treated cells, and lighter colors show the same plot for cells treated with lower concentrations. (b) Average mass plotted against time for 5-fluorouracil (5FU) treated MDA-MB-231 cells. Mass is normalized by the initial mass of each cell in the population. Dark gray shows the normalized mass of cells treated with 20 µM 5FU and lighter gray shows cells treated with 2 µM 5FU. Error bars show SEM. (c) MCF-7 response to 20 µM carboplatin (orange) versus DMSO control (blue) in 12 h bins shows an initially similar distribution that slowly begins to deviate over time. Solid lines represent the median of the distribution as a function of time. Dashed line shows SGR of zero. Individual data points shown within population distributions. (d) MDA-MB-231 response to 20 µM 5FU (gray) versus DMSO control (blue) in 12 h bins shows an initially similar distribution that slowly begins to deviate over time. Solid lines represent the median of the distribution as a function of time. Dashed line shows SGR of zero. Individual data points shown within population distributions.

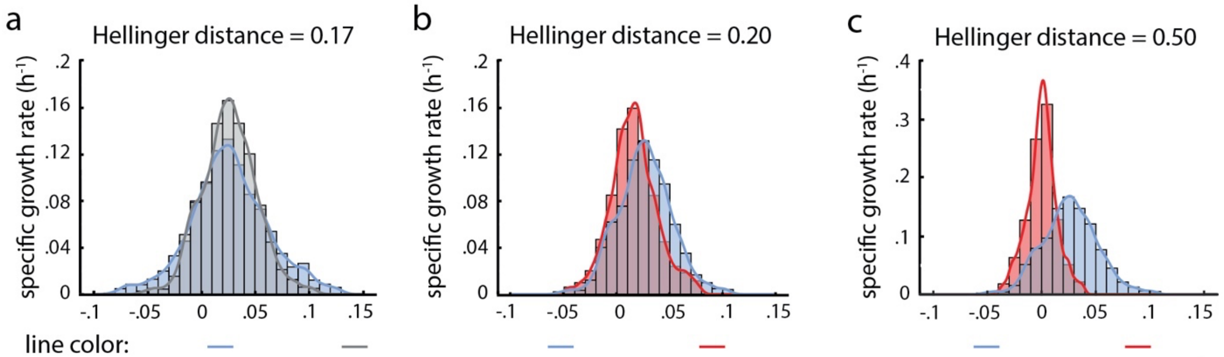

treatment:	DMSO	DMSO	DMSO	doxorubicin	DMSO	doxorubicin
concentration:	control	control	control	0.08 μM	control	0.4 μM
time:	28 h	58 h	28 h	28 h	28 h	28 h
mean:	0.0262 h^{-1}	0.0261 h^{-1}	0.0262 h^{-1}	0.0129 h^{-1}	0.0262 h^{-1}	0.00 h^{-1}
SD:	0.0256 h^{-1}	0.0359 h^{-1}	0.0256 h^{-1}	0.0239 h^{-1}	0.0256 h^{-1}	0.0138 h^{-1}

Supplementary Figure S9: Hellinger distance used to determine the statistical distance between drug treated and control treated groups. (a) The specific growth rate distributions for DMSO treated MDA-MB-231 cells shows a small change during the experiment setting the threshold for DMSO treated cells in this experiment. (b) Specific growth rate distribution of MDA-MB-231 cells treated with .08 μM of doxorubicin has a reduced mean and a Hellinger distance from the control greater than the threshold indicating a response. (c) Specific growth rate distribution of MDA-MB-231 cells treated with .4 μM of doxorubicin has a much greater Hellinger distance from the control due to the depression of both the mean and standard deviation of the distribution.

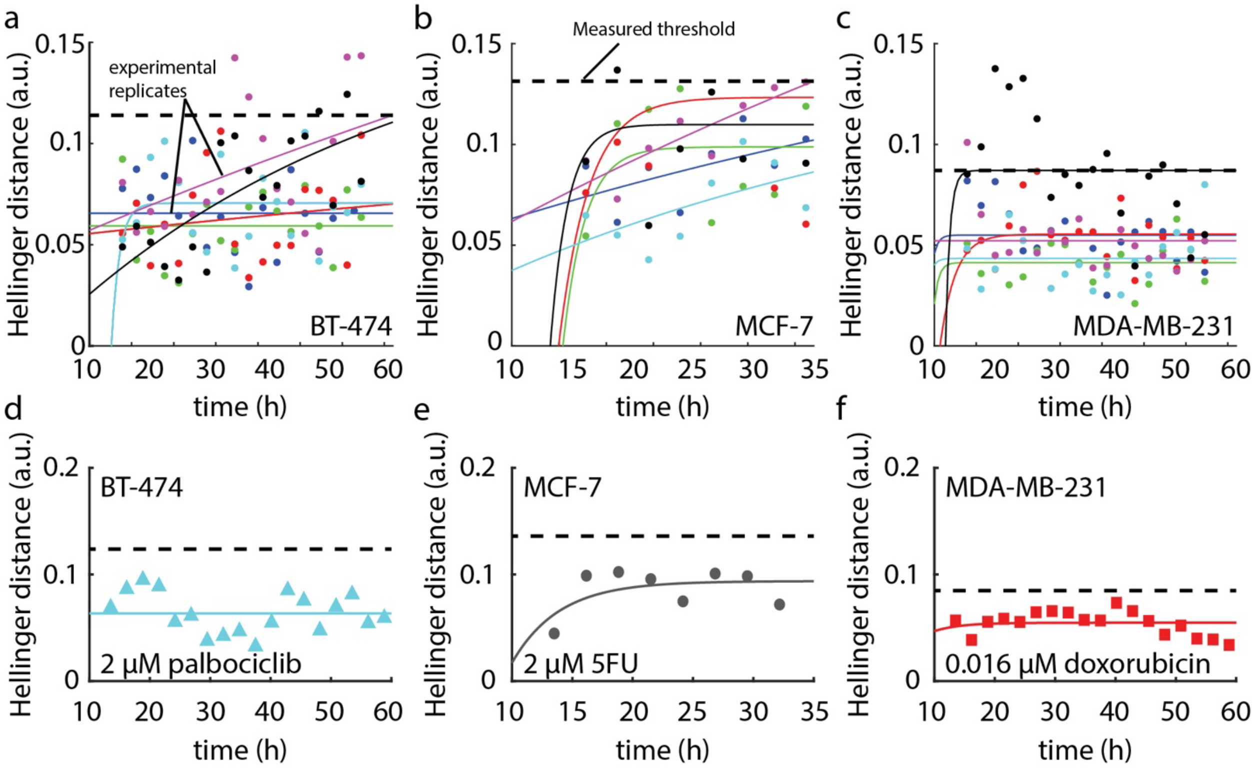

Supplementary Figure S10: Measuring Hellinger distance. (a-c) Hellinger distance over time between the DMSO treated cells and the untreated control shows that the maximum measured Hellinger distance is (a) 0.112 for BT-474, (b) 0.136 for MCF-7, (c) and 0.092 to determine the threshold Hellinger distance used to determine ToR. (d-f) Conditions that show no response never cross this threshold because the Hellinger distance between the treated group and the control is similar to the Hellinger distance between the two controls.

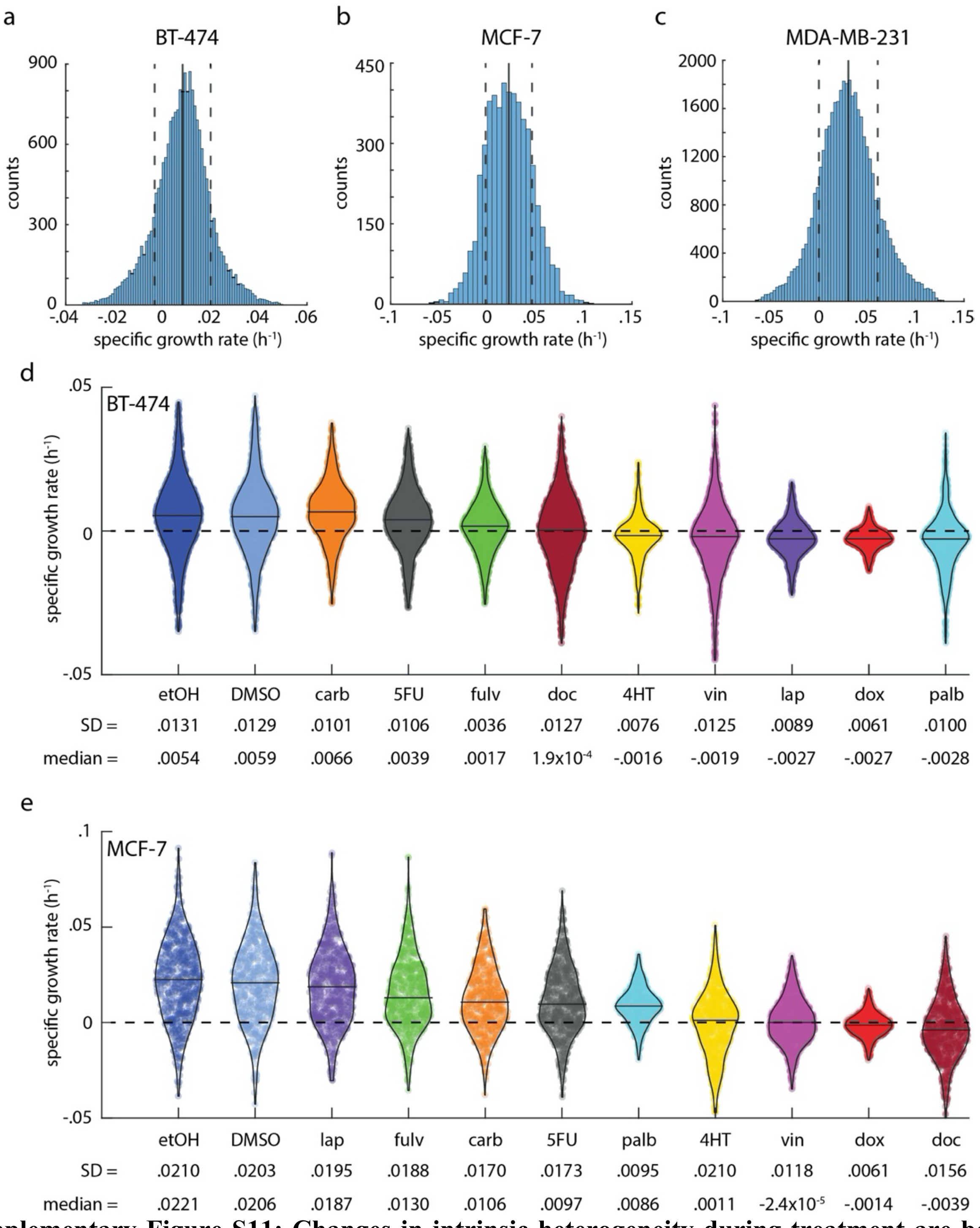

Supplementary Figure S11: Changes in intrinsic heterogeneity during treatment are both drug and cell-line dependent. (a-c) Each cell line exhibits baseline growth heterogeneity in the control population. Mean shown as vertical solid line, mean +/- standard deviation shown as dashed lines. (BT-474, *n* = 20,593; MCF-7, *n* = 5,218; MDA-MB-231, *n* = 42,559). (d) Growth rate distribution of BT-474 cells exposed to 20 μM of each indicated drug. Data is ordered from highest to lowest mean growth rate. (e) Growth rate distribution of MCF7 clusters exposed to 20 μM drug ordered from highest to lowest mean growth rate.

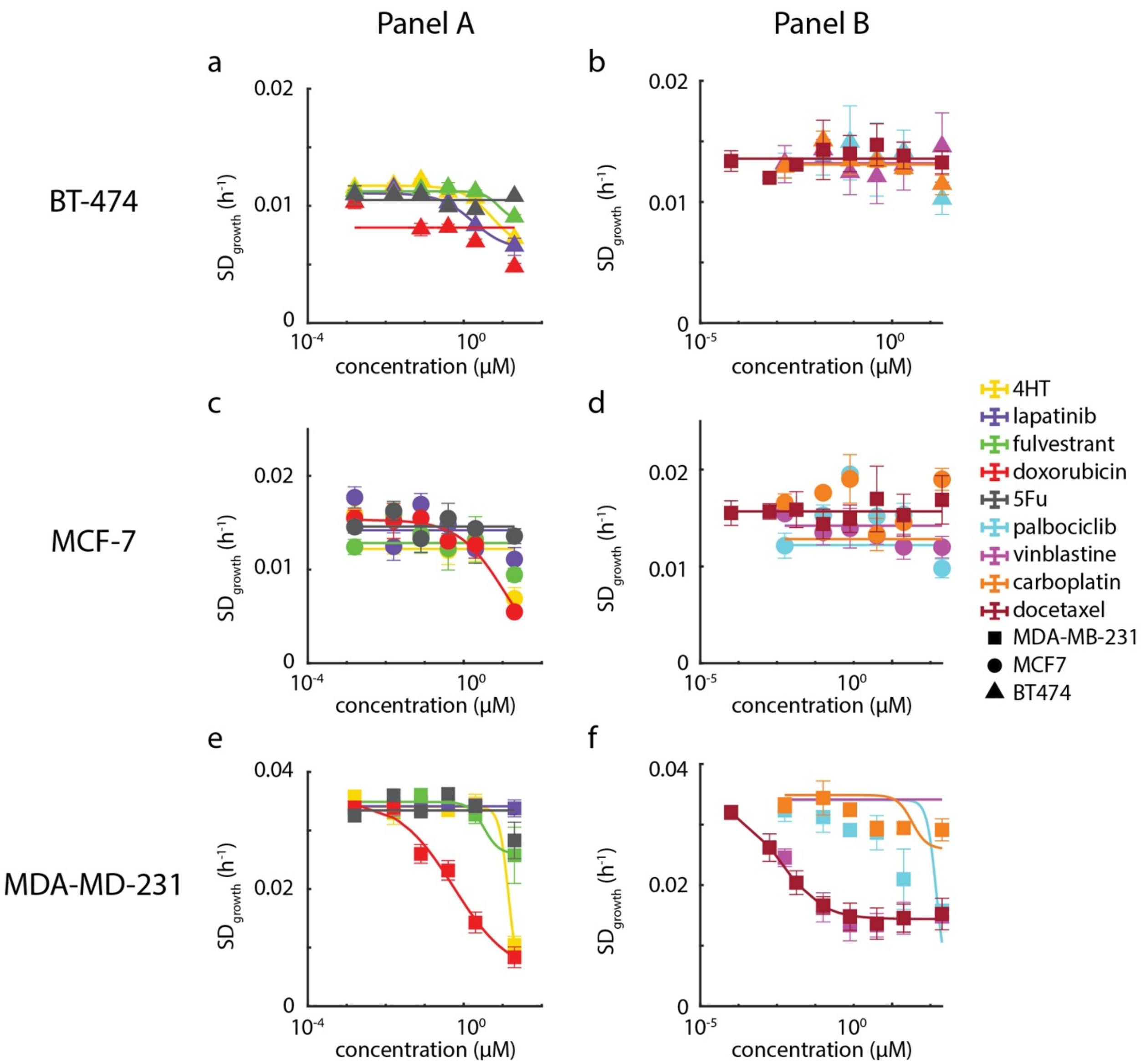

Supplementary Figure S12: Dose response of growth heterogeneity versus therapy concentration. Plots show standard deviation of 72 h cell cluster population specific growth rate as a function of drug concentration. Hill equation fit is only shown when there is a measurable response, as indicated when compared to a flat line fit using an F-test to reject the null hypothesis that the two fits are indistinguishable. Only a small fraction (8 out of 18 conditions) show a measurable change in population standard deviation at increasing drug concentration. Error bars show SEM.

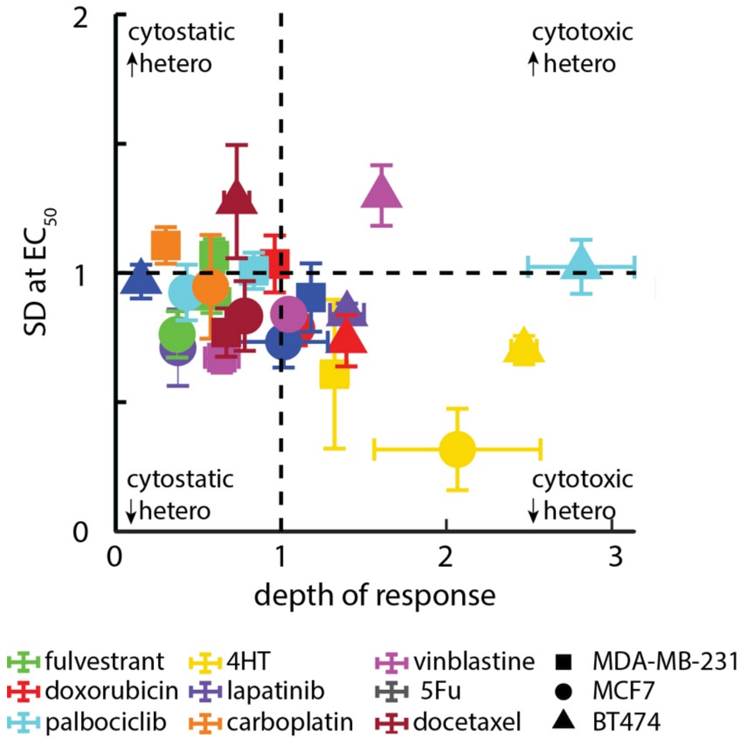

Supplementary Figure S13: Standard deviation at EC_{50} plotted against DoR. Horizontal dashed line divides fast responders from slow responders, vertical dashed line divides cytotoxic conditions from cytostatic. Error bars show SEM. Heterogeneity is abbreviated as hetero.

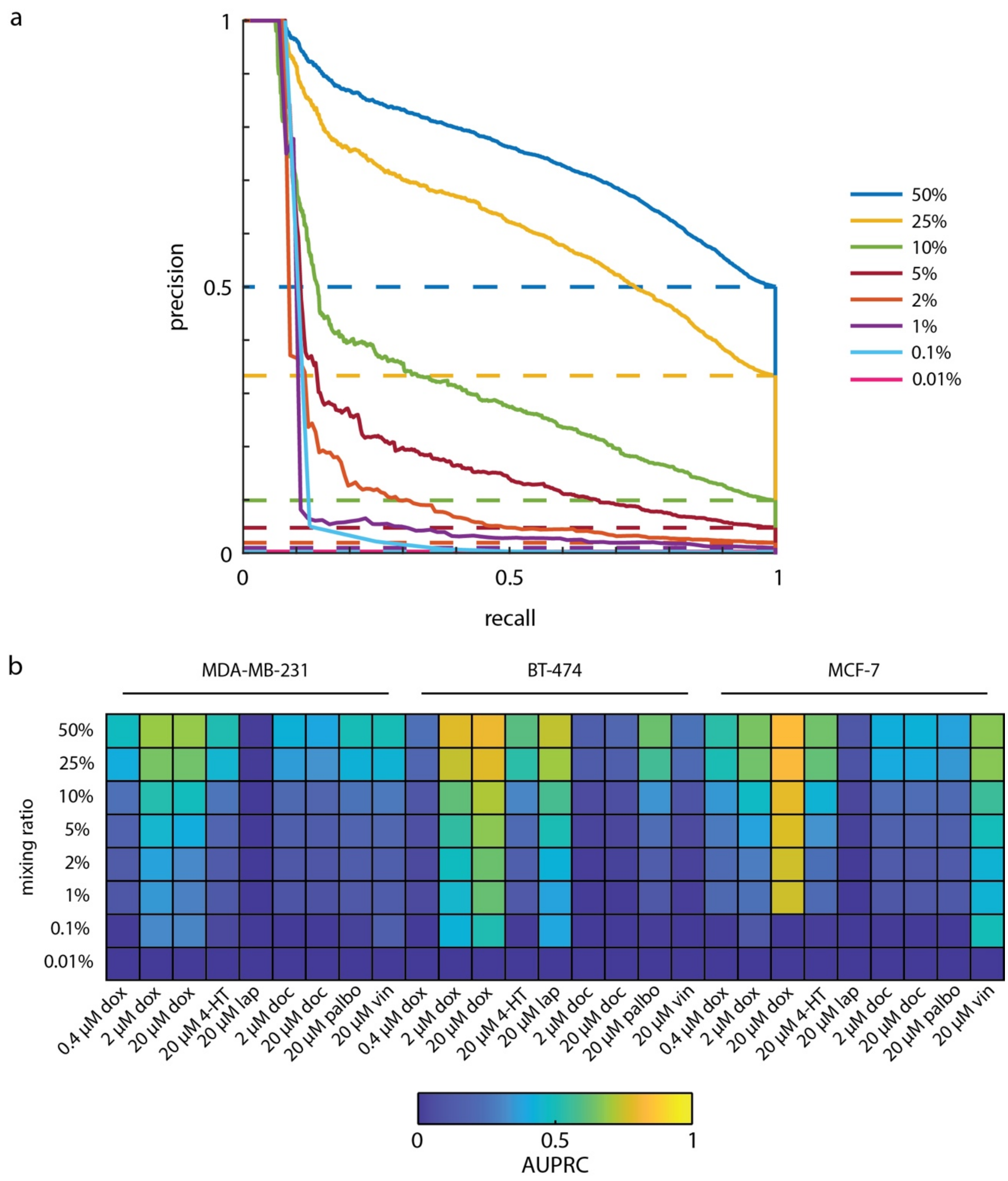

Supplementary Figure S14: Identifying resistant cells using specific growth rate. (a) Precision-recall curves for MDA-MB-231 cells treated with 2 µM doxorubicin mixed with control cells in ratios of 50%, 25%, 10%, 5%, 2%, 1%, 0.1%, and 0.01%. Horizontal line for each ratio represents no ability to distinguish the control cells. (b) Heat map shows a summary of precision-recall analysis for a range of responding conditions for all three cell lines.

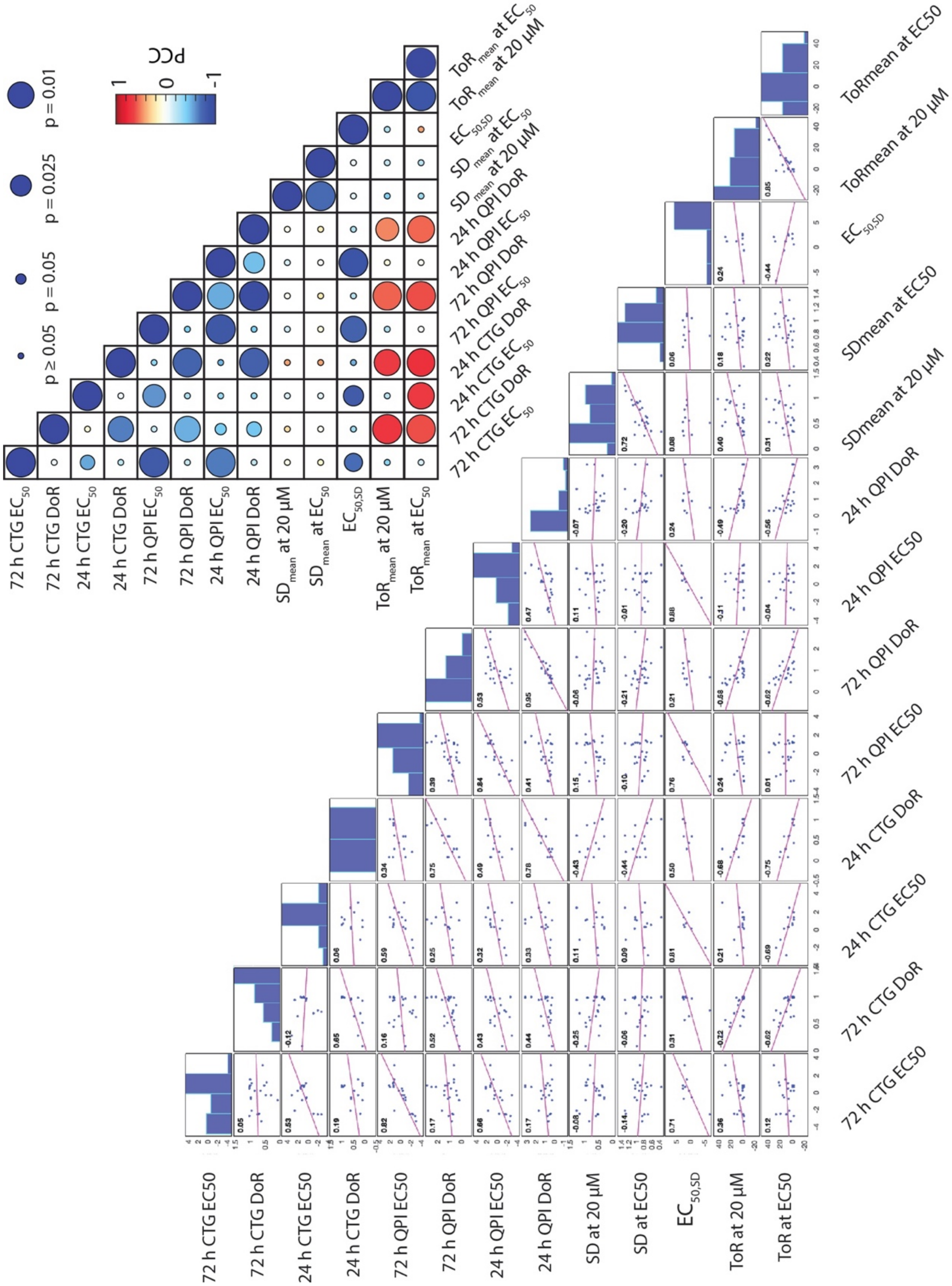

Supplementary Figure S15: Correlations between the drug response parameters measured using QPI demonstrate orthogonality between measurements. Correlation matrix measures the Pearson correlation coefficient and shows strong correlations as dark blue, negative correlations as red, and no correlation as white. The size of the circle shows the level of significance of the measured Pearson coefficient. Correlation plots show the data used to compute the correlation coefficient.

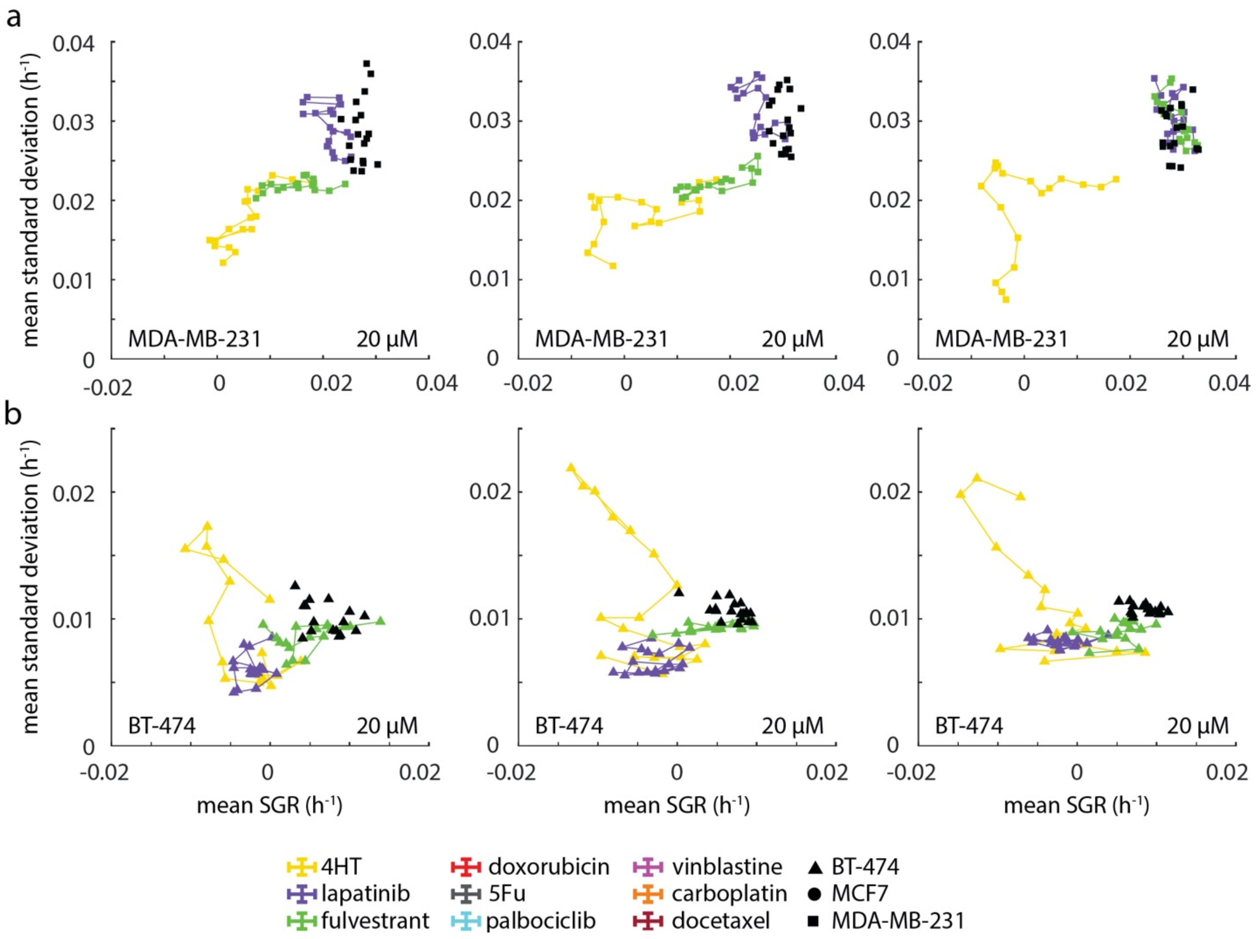

Supplementary Figure S16: Individual replicates of time parameterized SD vs SGR. (a) Plot of mean specific growth rate plotted against standard deviation in growth parameterized by time for MDA-MB-231 cells treated with drug panel A. Each track shows behavior from an individual experiment. (b) Plot of mean specific growth rate plotted against standard deviation in growth parameterized by time for BT-474 cells treated with drug panel A. Each track shows behavior from an individual experiment. Control cells are shown as a cluster of black points.

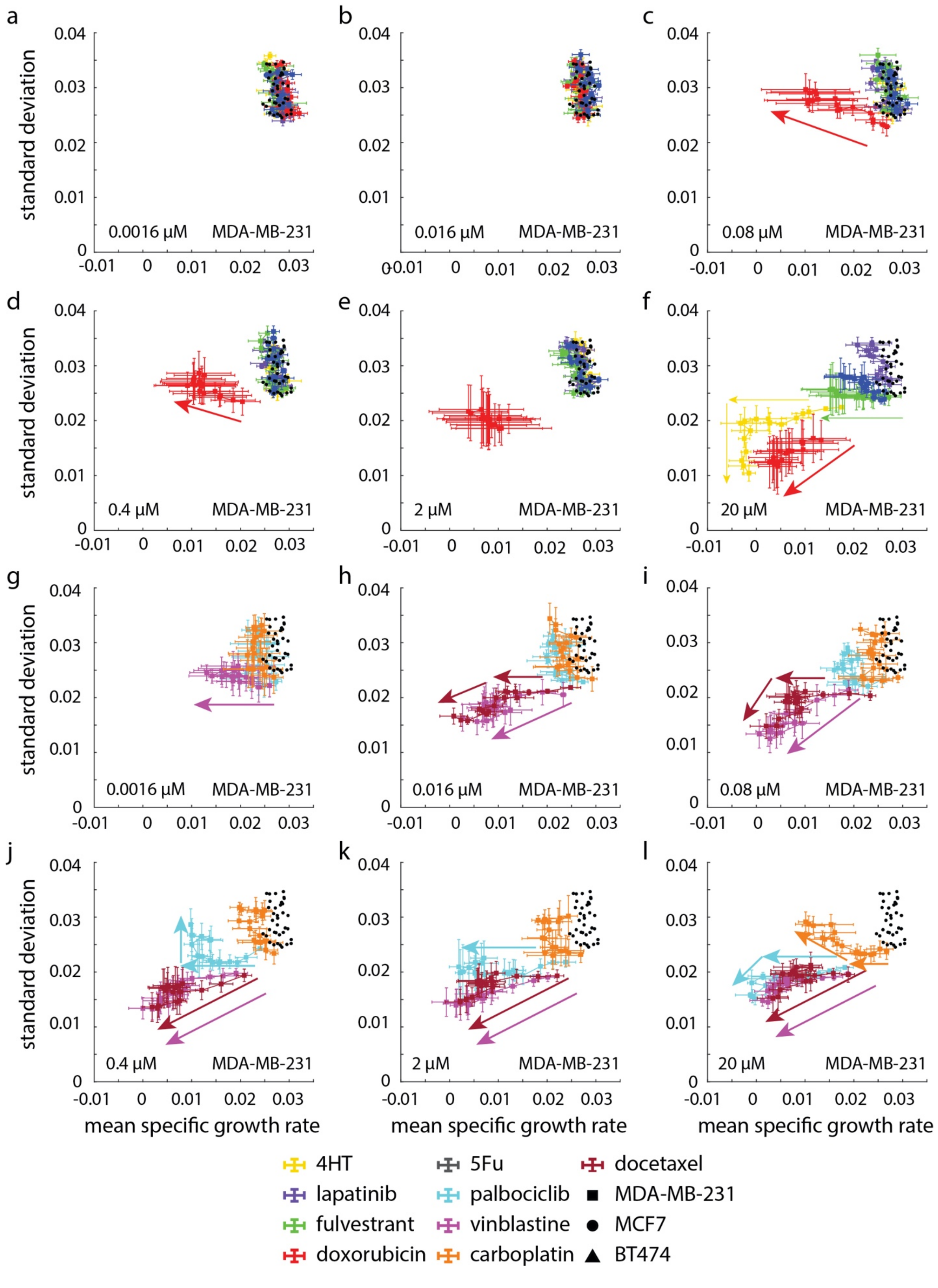
a
b
c
d
e
f
g
h
i
j
k
l
standard deviation
mean specific growth rate
0.0016 μM
0.016 μM
0.08 μM
0.4 μM
2 μM
20 μM
MDA-MB-231
4HT
lapatinib
fulvestrant
doxorubicin
5Fu
palbociclib
vinblastine
carboplatin
docetaxel
MDA-MB-231
MCF7
BT474

Supplementary Figure S17: Heterogeneity vs specific growth rate parameterized by time for MDA-MB-231 cells shows increase in heterogeneity in response to therapy. (a)-(b) MDA-MB-231 cells treated with drug panel A show no change in growth rate or heterogeneity over time at the lowest concentrations. At middle concentrations (c)-(e) doxorubicin shows a change in growth rate over time with little to no change in heterogeneity. (f) At the highest concentration, doxorubicin simultaneously decreases in growth rate and heterogeneity, while 4HT first decreases in growth rate and then decreases in heterogeneity. (g)-(h) At the lowest concentrations vinblastine and docetaxel start at a growth rate similar to the control and growth rate decreases over time with little change in heterogeneity. (h) At 0.08 μM MDA-MB-231 cells treated with both vinblastine and docetaxel decrease in growth rate and heterogeneity simultaneously. (j) At 0.4 μM, MDA-MB-231 cells treated with vinblastine and docetaxel decrease in growth rate and heterogeneity simultaneously, but cells treated with palbociclib first decrease in growth rate with little change in heterogeneity relative to the control. (k) At 2 μM, MDA-MB-231 cells treated with vinblastine and docetaxel decrease in growth rate and heterogeneity simultaneously, but cells treated with palbociclib decrease in growth rate with little change in heterogeneity. (l) At 20 μM, MDA-MB-231 cells treated with vinblastine and docetaxel decrease in growth rate and heterogeneity simultaneously, but cells treated with palbociclib first decrease in growth rate before decreasing slightly in heterogeneity. Carboplatin causes a decrease in growth rate with little effect on heterogeneity relative to the control. Arrows show forward direction in time. Error bars show SEM.

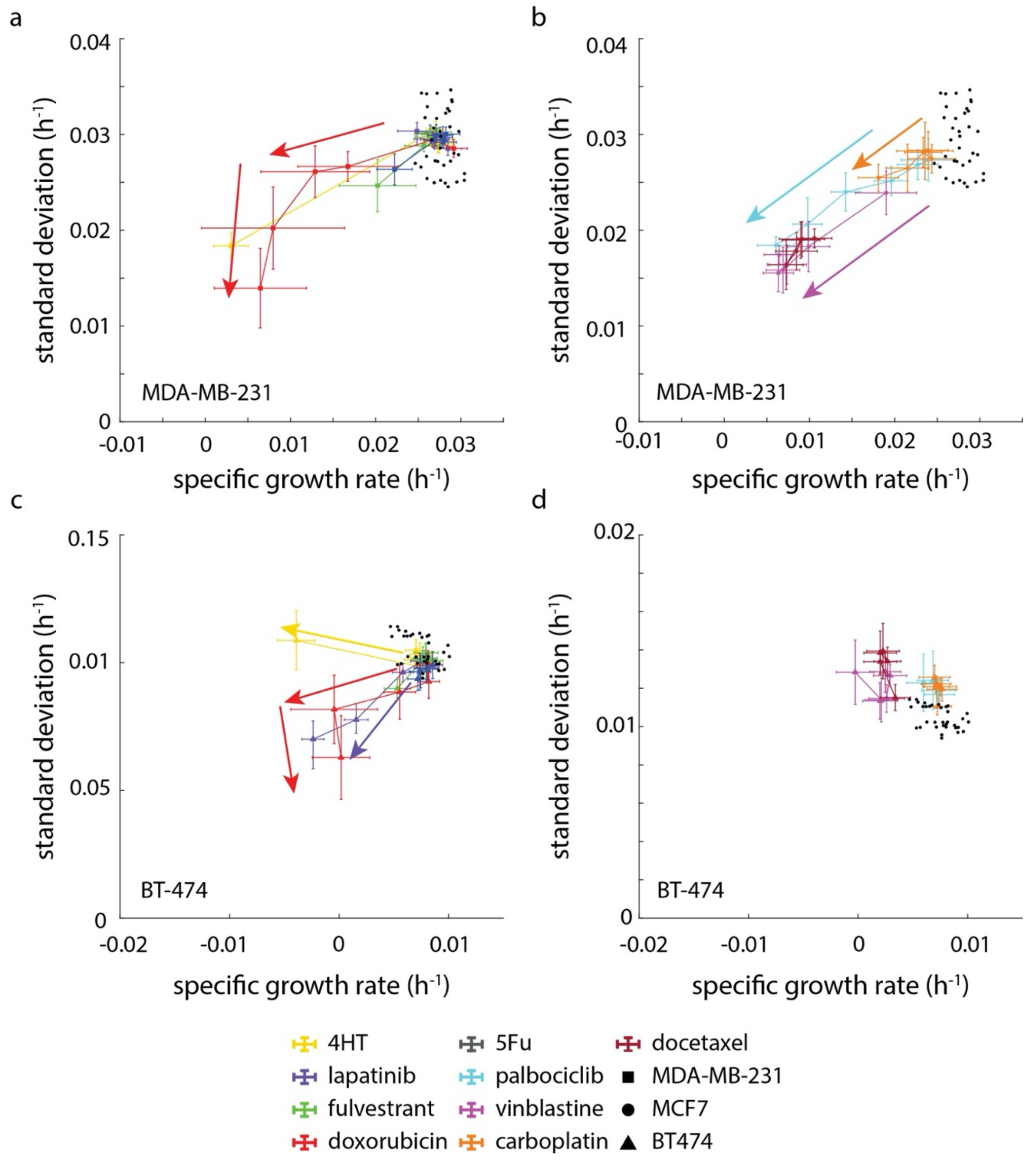

Supplementary Figure S18: Heterogeneity vs specific growth rate parameterized by concentration for MDA-MB-231 and BT-474 cells shows increase in heterogeneity in response to therapy. (a) As concentration increases doxorubicin treated MDA-MB-231 cells decrease in both growth rate and heterogeneity. (b) As concentration increases palbociclib, carboplatin, and vinblastine treated MDA-MB-231 cells decrease in growth rate and heterogeneity simultaneously. (c) BT-474 treated with doxorubicin, and lapatinib decrease in growth rate and heterogeneity, while 4HT treated cells decrease in growth rate at approximately constant standard deviation. (d) Doxorubicin and vinblastine treated BT-474 cells decrease in growth rate as concentration increases while heterogeneity remains constant. Arrows show direction of increasing concentration. Error bars show SEM.

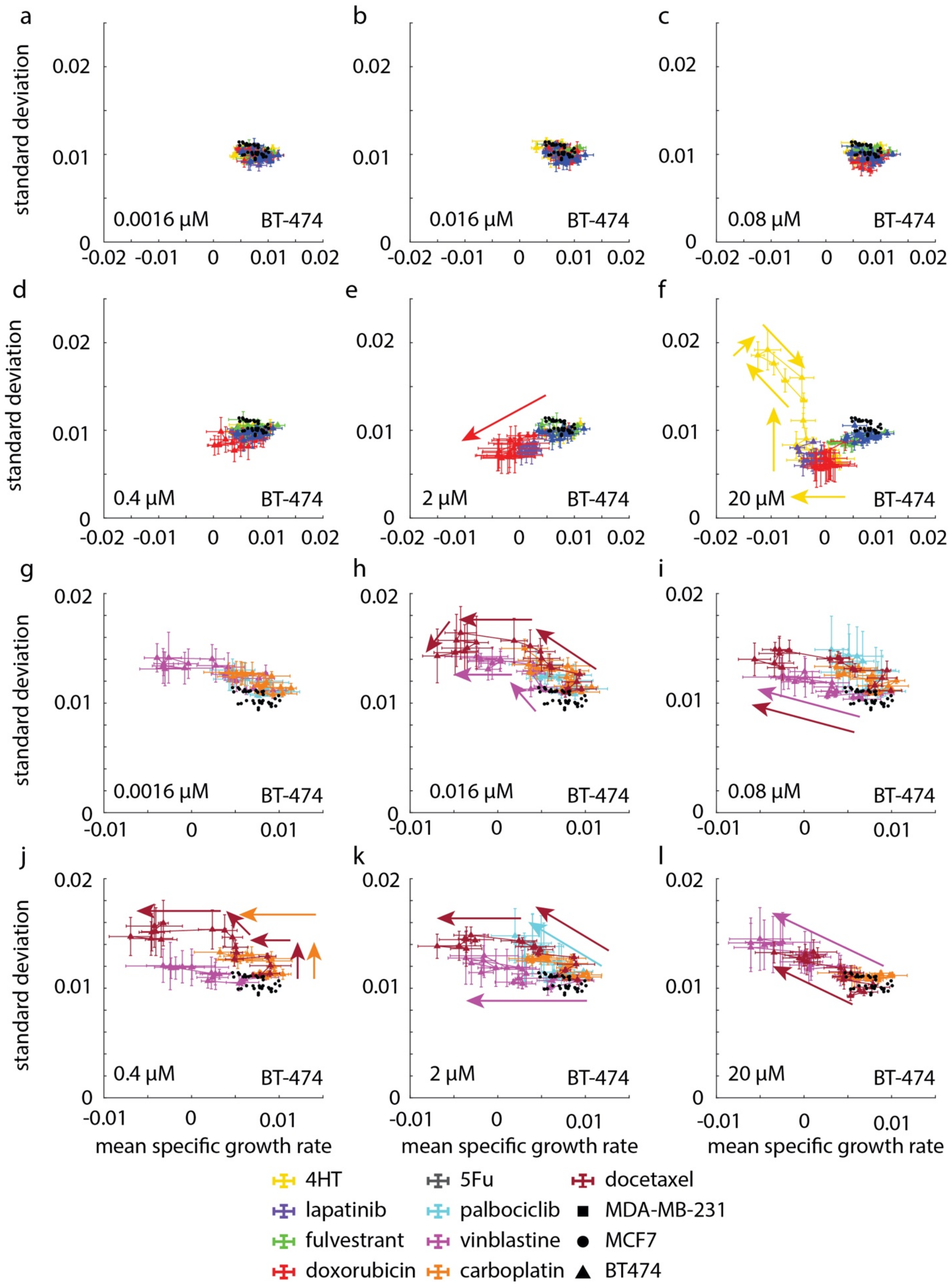
a
b
c
d
e
f
g
h
i
j
k
l
standard deviation
mean specific growth rate
0.0016 µM
0.016 µM
0.08 µM
0.4 µM
2 µM
20 µM
BT-474
0.02
0.01
0
-0.02
-0.01
0.01
4HT
lapatinib
fulvestrant
doxorubicin
5Fu
palbociclib
vinblastine
carboplatin
docetaxel
MDA-MB-231
MCF7
BT474

Supplementary Figure S19: Heterogeneity vs specific growth rate parameterized by time for BT-474 cells shows increase in heterogeneity in response to therapy. (a-d) At concentrations below the EC_{50} both standard deviation and the mean specific growth rate for drug treated populations are indistinguishable from the control (shown as black dots). (e) Near the EC_{50} concentration, the mean growth rate and heterogeneity of the population both decrease simultaneously. (f) In contrast, BT-474 cells respond to 4-hydroxy-tamoxifen by first experiencing reduced growth rate, and then a large spike in heterogeneity in response to the therapy. Only after the growth rate is further reduced does the heterogeneity of the population begin to decrease. (g) BT-474 responds to vinblastine at the lowest concentration (0.0016 µM) causing a decrease in growth rate at constant heterogeneity that is greater than the control. (h) BT-474 responds to both vinblastine and docetaxel at 0.016 µM causing a decreased growth rate and increased heterogeneity. (i) At a concentration of 0.08 µM, BT-474 cells respond with reduced growth rate and increased heterogeneity. (j) When treated with 0.4 µM of therapy, BT-474 cells respond to docetaxel with reduced growth rate and increased heterogeneity but respond to vinblastine with reduced growth rate at the same level of heterogeneity as the control. (k) At 2 µM of treatment concentration population heterogeneity is increased and growth rate decreases in response to both docetaxel and palbociclib. (l) At 20 µM concentration the growth rate of docetaxel and vinblastine treated cells decreases, while the heterogeneity for both populations increases. Arrows show forward direction in time. Error bars show SEM.
