# Supplementary material for: Multiparametric quantitative phase imaging for real-time, single cell, drug screening in breast cancer
Source: Commun Biol. 2022 Aug 8;5:794. doi: 10.1038/s42003-022-03759-1 (PMC9360018; doi:10.1038/s42003-022-03759-1)
Supplement: Supplementary file 3 — Description of Additional Supplementary Files [file 42003_2022_3759_MOESM3_ESM.pdf]

Description of Additional Supplementary Files

File name: Supplementary Data 1
Description: All fitting parameters for all conditions and all experiments.

File name: Supplementary Data 2
Description: All derived response parameters from all conditions and experiments.

File name: Supplementary Movie S1
Description: Video of entire field of view of individual MDA-MB-231 cells treated with DMSO proliferating throughout the duration of the experiment.

File name: Supplementary Movie S2
Description: Video of entire field of view of clusters of BT-474 cells treated with DMSO proliferating throughout the duration of the experiment.

File name: Supplementary Movie S3
Description: Video of entire field of view of clusters of MCF7 cells treated with DMSO proliferating throughout the duration of the experiment.

File name: Supplementary Movie S4
Description: Video of individual MDA-MB231 cell treated with DMSO.

File name: Supplementary Movie S5
Description: Video of a cluster of BT-474 cells treated with DMSO.

File name: Supplementary Movie S6
Description: Video of a cluster of MCF7 cells treated with DMSO.

File name: Supplementary Movie S7
Description: Video of individual MDA-MB231 cell treated with 2 µM doxorubicin.

File name: Supplementary Movie S8
Description: Video of a cluster of BT-474 cells treated with 2 µM doxorubicin.

File name: Supplementary Movie S9
Description: Video of a cluster of MCF7 cells treated with 20 µM fulvestrant.

File name: Supplementary Movie S10
Description: Video of MDA-MB-231 cell growing despite treatment with 20 µM docetaxel.
