# Supplementary material for: Multiparametric quantitative phase imaging for real-time, single cell, drug screening in breast cancer
Source: Commun Biol. 2022 Aug 8;5:794. doi: 10.1038/s42003-022-03759-1 (PMC9360018; doi:10.1038/s42003-022-03759-1)
Supplement: Supplementary file 16 — Reporting Summary [file 42003_2022_3759_MOESM16_ESM.pdf]

nature portfolio

Corresponding author(s): Thomas A. Zangle

Last updated by author(s): June 24, 2022

Reporting Summary

Nature Portfolio wishes to improve the reproducibility of the work that we publish. This form provides structure for consistency and transparency in reporting. For further information on Nature Portfolio policies, see our Editorial Policies and the Editorial Policy Checklist.

Statistics

For all statistical analyses, confirm that the following items are present in the figure legend, table legend, main text, or Methods section.

n/a	Confirmed	
☐	☒	The exact sample size (n) for each experimental group/condition, given as a discrete number and unit of measurement
☐	☒	A statement on whether measurements were taken from distinct samples or whether the same sample was measured repeatedly
☐	☒	The statistical test(s) used AND whether they are one- or two-sided<br>*Only common tests should be described solely by name; describe more complex techniques in the Methods section.*
☐	☒	A description of all covariates tested
☐	☒	A description of any assumptions or corrections, such as tests of normality and adjustment for multiple comparisons
☐	☒	A full description of the statistical parameters including central tendency (e.g. means) or other basic estimates (e.g. regression coefficient) AND variation (e.g. standard deviation) or associated estimates of uncertainty (e.g. confidence intervals)
☐	☒	For null hypothesis testing, the test statistic (e.g. F, t, r) with confidence intervals, effect sizes, degrees of freedom and P value noted<br>*Give P values as exact values whenever suitable.*
☒	☐	For Bayesian analysis, information on the choice of priors and Markov chain Monte Carlo settings
☒	☐	For hierarchical and complex designs, identification of the appropriate level for tests and full reporting of outcomes
☒	☐	Estimates of effect sizes (e.g. Cohen's d, Pearson's r), indicating how they were calculated

Our web collection on statistics for biologists contains articles on many of the points above.

Software and code

Policy information about availability of computer code

Data collection	A custom quantitative phase microscope was used to collect the data used in this study. The microscope was programmed using python and the parameters used were clearly described in the manuscript (e.g. illumination setup, exposure time on camera, etc.)
Data analysis	Data analysis was performed using custom Matlab code that is available on the lab Github page at the following URL: https://github.com/Zangle-Lab/MultiparametricQPI

For manuscripts utilizing custom algorithms or software that are central to the research but not yet described in published literature, software must be made available to editors and reviewers. We strongly encourage code deposition in a community repository (e.g. GitHub). See the Nature Portfolio guidelines for submitting code & software for further information.

Data

Policy information about availability of data

All manuscripts must include a data availability statement. This statement should provide the following information, where applicable:

- Accession codes, unique identifiers, or web links for publicly available datasets
- A description of any restrictions on data availability
- For clinical datasets or third party data, please ensure that the statement adheres to our policy

All data to reproduce the results of this study is publicly available on figshare using the following doi: 10.6084/m9.figshare.16840435

Field-specific reporting

Please select the one below that is the best fit for your research. If you are not sure, read the appropriate sections before making your selection.

☒ Life sciences ☐ Behavioural & social sciences ☐ Ecological, evolutionary & environmental sciences

For a reference copy of the document with all sections, see nature.com/documents/nr-reporting-summary-flat.pdf

Life sciences study design

All studies must disclose on these points even when the disclosure is negative.

Sample size	We optimized our experiments for the largest possible sample size, by selecting positions with the most number of cells and performing each condition in triplicate within each experiment as well as repeating each experiment three times.
Data exclusions	Outliers were removed from the dataset using the approach outlined in the methods section of the manuscript. Briefly, we used a linear regression to determine the growth rate of individual cells and using the standard error and normalized slope, outliers (defined as 3 median absolute deviation from the mean) were then removed from the analysis.
Replication	Three replicates for each condition were studied in each experiment and three separate experiments performed on random days to validate reproducibility of the data.
Randomization	Cells were chosen at random and imaging locations containing cells were selected to obtain the greatest the number of individual cells per experiment.
Blinding	The code for data analysis was written using preliminary experiments, blind to the potential results of the experiments ultimately described in this study. This allowed for the analysis to unbiased in regards to the outcome of the study.

Reporting for specific materials, systems and methods

We require information from authors about some types of materials, experimental systems and methods used in many studies. Here, indicate whether each material, system or method listed is relevant to your study. If you are not sure if a list item applies to your research, read the appropriate section before selecting a response.

Materials & experimental systems

n/a	Involved in the study	
☒	☐	Antibodies
☐	☒	Eukaryotic cell lines
☒	☐	Palaeontology and archaeology
☒	☐	Animals and other organisms
☒	☐	Human research participants
☒	☐	Clinical data
☒	☐	Dual use research of concern

Methods

n/a	Involved in the study	
☒	☐	ChIP-seq
☒	☐	Flow cytometry
☒	☐	MRI-based neuroimaging

Eukaryotic cell lines

Policy information about cell lines

Cell line source(s)	ATCC
Authentication	Cell lines were newly acquired from ATCC or re-analyzed by short tandem repeat (ATR) analysis.
Mycoplasma contamination	The Zangle lab routinely monitors all active cell cultures for mycoplasma infection by DAPI staining.
Commonly misidentified lines (See ICLAC register)	MCF-7 was used in this study as a widely studied representative of the ER+ breast cancer subtype.
